# Tracing the Neural Carryover Effects of Interpersonal Anger on Resting-State fMRI in Men and Their Relation to Traumatic Stress Symptoms in a Subsample of Soldiers

**DOI:** 10.3389/fnbeh.2017.00252

**Published:** 2017-12-20

**Authors:** Gadi Gilam, Adi Maron-Katz, Efrat Kliper, Tamar Lin, Eyal Fruchter, Ron Shamir, Talma Hendler

**Affiliations:** ^1^The Tel Aviv Center for Brain Function, Wohl Institute for Advanced Imaging, Tel Aviv Sourasky Medical Center, Tel Aviv, Israel; ^2^School of Psychological Sciences, Tel-Aviv University, Tel Aviv, Israel; ^3^Sackler Faculty of Medicine, Tel-Aviv University, Tel Aviv, Israel; ^4^Blavatnik School of Computer Science, Tel-Aviv University, Tel Aviv, Israel; ^5^Division of Mental Health, Israeli Defense Force Medical Corp, Haifa, Israel; ^6^Sagol School of Neuroscience, Tel-Aviv University, Tel Aviv, Israel

**Keywords:** anger, rumination, recovery, stress, PTSD, amygdala, IFG, fMRI

## Abstract

Uncontrolled anger may lead to aggression and is common in various clinical conditions, including post traumatic stress disorder. Emotion regulation strategies may vary with some more adaptive and efficient than others in reducing angry feelings. However, such feelings tend to linger after anger provocation, extending the challenge of coping with anger beyond provocation. Task-independent resting-state (rs) fMRI may be a particularly useful paradigm to reveal neural processes of spontaneous recovery from a preceding negative emotional experience. We aimed to trace the carryover effects of anger on endogenous neural dynamics by applying a data-driven examination of changes in functional connectivity (FC) during rs-fMRI between before and after an interpersonal anger induction (*N* = 44 men). Anger was induced based on unfair monetary offers in a previously validated decision-making task. We calculated a common measure of global FC (gFC) which captures the level of FC between each region and all other regions in the brain, and examined which brain regions manifested changes in this measure following anger. We next examined the changes in all functional connections of each individuated brain region with all other brain regions to reveal which connections underlie the differences found in the gFC analysis of the previous step. We subsequently examined the relation of the identified neural modulations in the aftermath of anger with state- and trait- like measures associated with anger, including brain structure, and in a subsample of designated infantry soldiers (*N* = 21), with levels of traumatic stress symptoms (TSS) measured 1 year later following combat-training. The analysis pipeline revealed an increase in right amygdala gFC in the aftermath of anger and specifically with the right inferior frontal gyrus (IFG).We found that the increase in FC between the right amygdala and right IFG following anger was positively associated with smaller right IFG volume, higher trait-anger level and among soldiers with more TSS. Moreover, higher levels of right amygdala gFC at baseline predicted less reported anger during the subsequent anger provocation. The results suggest that increased amygdala-IFG connectivity following anger is associated with maladaptive recovery, and relates to long-term development of stress symptomatology in a subsample of soldiers.

## Introduction

“Do not mix anger with profusion and set them before your guests. Profusion makes its way through the body, and is quickly gone: but anger, when it hath penetrated the soul, abides for a long time.”

*-Epictatus*

Anger is experienced on a daily basis and mostly during or following social interactions (Averill, 1983; Baumeister et al., 1990), possibly leading to aggression and violence towards the environment (Anderson and Bushman, 2002; Rosell and Siever, 2015). Excessive anger may also have negative consequences on one’s health, wellbeing and social rapport (Johnson, 1990; Williams, 2010). Moreover, uncontrolled anger is prevalent in numerous psychopathological conditions (Novaco, 2010), such as in post-traumatic stress disorder (PTSD; Olatunji et al., 2010). The importance of regulating anger and adapting it to socially accepted norms is thus unequivocal (Davidson, 2000; Gilam and Hendler, 2015). Notably, as Epictatus’s quotation illustrates, the challenge of coping with and recovering from anger extends beyond the termination of the anger-inducing provocation since feelings of anger tend to linger and outlast the provocation itself (Potegal, 2010). In fact, individuals with a chronic tendency to be angry (i.e., high in trait-anger) have difficulties in disengaging from such lingering anger, paralleled by impaired recruitment of regulatory resources (Sukhodolsky et al., 2001; Wilkowski and Robinson, 2010).

There are various emotion regulation strategies one may recruit to cope with anger, some more adaptive than others. For example, cognitive reappraisal, which refers to the reinterpretation of an emotional event, was shown to be effective in reducing angry feelings (e.g., Ray et al., 2008; Szasz et al., 2011). On the other hand, angry rumination, which refers to recurrent thought patterns on causes and consequences of the angering episode, was shown to intensify and prolong the experience of anger and increase subsequent aggression (e.g., Bushman et al., 2005; Pedersen et al., 2011). Indeed, rumination is considered a maladaptive regulatory response, common also in PTSD patients (Michael et al., 2007).

The neural bases of emotion regulation generally engages prefrontal cortex (PFC) regions such as the ventro-medial PFC (vmPFC) and inferior frontal gyrus (IFG) which exert control over emotion reactivity regions such as the amygdala and insula (Diekhof et al., 2011; Frank et al., 2014; Etkin et al., 2015). In the context of an anger experience, it was previously demonstrated that while reappraisal and rumination similarly engaged the activation of such regions, differences between these strategies emerged only in connectivity (Fabiansson et al., 2012). Specifically, there was a positive correlation between the IFG and both amygdala and thalamus during rumination but not reappraisal. However, emotion regulation was explicitly instructed in that study, limiting our understanding of naturalistic anger regulation. We recently developed a modified Ultimatum Game in which participants faced unfair anger-inducing monetary offers infused with verbal provocations by a competitor (Gilam et al., 2015). We demonstrated that vmPFC activation and posterior insula-medial thalamus connectivity modulated angry feelings leading to increased acceptance of unfair offers and thus gaining more money throughout the game, providing a neural model for spontaneous anger regulation. In the current study, we aimed to trace the changes in spontaneous neural processing in the aftermath of an angering episode, assuming this may shed light on endogenous regulatory processes enabling recovery from such turmoil. This may also reveal if recovering from anger in its aftermath engages similar or different neural processes as regulating anger during on-going anger provocation and potentially inform efforts to mitigate the negative implications of anger on people’s lives.

The resting-state (rs) fMRI paradigm in which participants’ brain is scanned while they let their thoughts wander without any instruction (Gruberger et al., 2011) may be particularly relevant to trace changes in spontaneous neural dynamics related to an immediately preceding emotional experience. A few previous studies adopted such an approach in regards to an induced emotional experience, mostly revealing increased neural coupling between prefrontal, limbic and paralimbic brain regions including the medial PFC, amygdala, cingulate and insular cortices, some of which were associated with sustainment of the emotional experience (e.g., Harrison et al., 2008; van Marle et al., 2010; Veer et al., 2011; Schultz et al., 2012; Vaisvaser et al., 2013; Maron-Katz et al., 2016; Clemens et al., 2017). In some of these studies a data-driven analysis was used instead of a seed-based analysis, having the benefit of being independent of any prior hypothesis and of an unbiased identification of brain regions in the entire brain (e.g., Harrison et al., 2008; Maron-Katz et al., 2016).

To reveal neural dynamics of an angering experience beyond its immediate occurrence, we used a data-driven analysis to examine changes in rs-functional connectivity (FC) from before to after an interpersonal angering experience. At first we performed a whole-brain analysis using a measure of global FC (gFC) which captures the level of FC between each region and all other regions in the brain, and examined which brain regions manifested changes in gFC following anger. This allowed assessing regional changes in gFC. GFC has been suggested to reflect the level of neural integration of a certain brain region and thus potentially having a role in coordinating cognition and behavior (Cole et al., 2010, 2012). Therefore, changes in gFC following anger might reflect processes associated with recovery from the emotional experience. We next used the identified brain regions in a secondary analysis in which we examined the changes in all functional connections of each individuated brain region with all other brain regions to reveal which connections underlie the differences found in the gFC analysis of the previous step.

Participants were those who underwent the anger-infused Ultimatum Game (UG) mentioned above. Thus, to examine if the identified rs-FC modulations associated with participants’ anger experience and reaction during provocation, we tested whether the identified modulations, either in gFC or in specific connections, corresponded to participants’ self-reported angry feelings and their total monetary gain accumulated throughout the game. While these represent state-like measures of anger, we also questioned whether trait-like measures of anger corresponded to any of the identified modulations. In fact it was previously demonstrated that trait-rumination levels corresponded to gray matter volume in the IFG and cingulate (Kühn et al., 2012). We therefore tested the correspondence of both trait-anger and gray matter volume in the same brain regions in which the rs-FC modulations were identified.

Lastly, our sample consisted of newly recruited infantry soldiers from a unit in the Israeli Defense Forces (IDF) and civil-service volunteers. Since the specific infantry unit recruits only male soldiers, participants in this study were only male. The soldiers were at the beginning of an intense combat-training period of 1 year which was subsequently shown to increase traumatic stress symptoms (TSS) levels (Gilam et al., 2017). The civilians had no change in TSS along a similar period of civil-service. Since anger dysregulation and rumination are characteristic of PTSD patients (Michael et al., 2007; Olatunji et al., 2010), we aimed to examined whether the identified rs-FC modulations identified in the entire sample at the beginning of their respective programs could be predictive of TSS levels in soldiers towards the end of combat-training, possibly linking neural processes of recovery from anger to later development of stress symptomatology.

## Materials and Methods

### Participants

Our sample consisted of 60 male participants (age = 18.62 ± 0.88, mean ± SD) that underwent the anger-infused UG task as previously reported (Gilam et al., 2015). All participants completed secondary education, had no reported history of psychiatric or neurological disorders, no current use of psychoactive drugs and normal or corrected-to-normal vision. Due to MRI malfunctions rs-fMRI data was unavailable for 11 participants, and an additional five were discarded due to excessive head movements (>2 mm/2°). Therefore the final sample for rs-fMRI analyses consisted of 44 participants. Twenty-nine of these participants were newly recruited soldiers designated to an infantry unit in the IDF, while the other 15 were pre-army civil-service volunteers. Sample size was based on the number of soldiers and civilians willing to volunteer as participants. All participants were at the beginning of their respective programs and since there were no differences between soldiers and civilians in any measures of the anger induction task they were collapsed as one group. For example, no differences were found in behavior in the modified UG (*p* = 0.22), emotion ratings (*p* = 0.61), physiological arousal (*p* = 0.58), neural activations (e.g., vmPFC *p* = 0.38), trait anger (*p* = 0.15) and stress symptoms (*p* = 0.51; Gilam et al., 2015, 2017). All participants provided written informed consent and the study was approved by the Institutional Ethics Committee of the Tel-Aviv Sourasky Medical Center and of the IDF Medical Corps in accordance with the Helsinki Declaration.

### Procedure

Two 6-min rs-scans with eyes open on a fixation cross were recorded immediately before (rest1) and after (rest2) an interpersonal anger-induction task previously extensively reported (Gilam et al., 2015). Briefly, participants played an anger-infused version of the Ultimatum Game in which they had to agree on how to split a sum of money between themselves and another putative player. The game consisted of 10 such rounds and after each round players verbally negotiated between them, while participants were led to believe that monetary offers they received were decided in real time and influenced by their bargaining. Anger was induced by a predefined sequence of mostly unfair monetary divisions offered by the putative player, who was actually a professional actor trained with scripted improvisations to further intensify the angry experience during negotiations by incorporating personal insults, violating norms of conduct and direct confrontations regarding the game. This manipulation reflected the importance of embedding social interactions when investigating emotional experiences (Gilam and Hendler, 2016). Before entering the scanner participants filled out a trait-anger questionnaire and post-scan they reported on their emotional experience in relation to the game.

### Behavioral Measures

*Trait-Anger* was assessed using the gold-standard State-Trait Anger Expression Inventory (Spielberger and Sydeman, 1994) and comprised 10 items rated on a 4-point frequency scale from 1 (not at all) to 4 (very much) related to the frequency of angry feelings experienced over time. Trait-Anger was calculated as the sum score of these items and showed good internal consistency (Cronbach’s *α* = 0.74).

*Total-Gain* was calculated as the sum of money accumulated throughout the entire game by accepting offers and used as an objective measure of individual differences reflecting the final outcome of the anger-infused Ultimatum Game. Rejecting more offers and gaining less money is associated with aggressive reactions while accepting more offers and gaining more money is associated with anger-coping conciliatory reactions.

*Angry feelings were* assessed based on an iterated version of the Geneva Emotion Wheel (GEW; Scherer, 2005) scheme which was used to obtain subjective reports of the emotional experience during the anger-infused game, on a round-by-round basis and in accordance with participants’ actual decisions. Rating was performed on a 7-point intensity scale from 0 (none) to 6 (very high), in relation to how they felt in that exact period during the actual game. An anger cluster of emotions, which includes Anger, Hostility, Contempt and Disgust, was the highest reported cluster of emotions, compared to all other GEW clusters of emotions (positive clusters including Pride, Elation, Happiness, Satisfaction, Relief, Hope, Interest and Surprise, and an additional negative cluster including Shame/Guilt, Boredom, Sadness and Anxiety; Gilam et al., 2015). Indeed as previously noted, anger was the highest reported emotion from all emotion categories used in the GEW (*p* < 0.05 with Tukey’s correction; uncorrected only for hostility and contempt). Importantly, the other negative affect related emotion categories were unaffected by the manipulation nor explained any variance in behavior. We could also show differences in this anger-cluster measure by types of provocations in the task (fair/unfair offers) as well as showing an escalation along the task. This supported that our anger induction was not a mere general negative mood induction. Here we used the average reported emotions for all periods and all rounds of the game in the anger cluster as the measure of angry feelings of each individual during the game.

*Traumatic stress-symptoms* (TSS) were quantified using the Post-Traumatic Stress Disorder Check-List—Military (PCL-M; Weathers et al., 1991; Spoont et al., 2015) questionnaire which assess stress-symptoms experienced specifically in relation to military experiences. Respondents rate each of 17 stress-symptoms items on a 5-point frequency scale from 1 (not at all) to 5 (extremely), indicating the extent to which they have experienced a specific symptom during the past month of military service. This measure was evaluated towards the end of combat-training and was available for 21 of the 29 soldiers initially recruited to the experiment. As previously indicated in the literature (Novaco and Chemtob, 2002; Jakupcak et al., 2007), a potential methodological confound may exist in correlations between anger related measures and TSS measures since arousal, anger and physical reactions are all anger concomitants as well as being symptoms of post traumatic stress. To avoid circularity between measures and refute this possible confound we removed these symptoms’ items (#5, #14, #16 and #17) from the PCL-M score (Gilam et al., 2017).

### MRI Data Acquisition

Brain imaging was performed by a GE 3T Signa Excite scanner using an 8-channel head coil at the Wohl Institute for Advanced Imaging, Tel-Aviv Sourasky Medical Center. Functional whole-brain scans were performed with gradient echo-planar imaging (EPI) sequence of functional T2*-weighted images (TR/TE = 3000/35 ms; flip angle = 90°; FOV = 200 × 200 mm; slice thickness = 3 mm; no gap; 39 interleaved top-to-bottom axial slices per volume; in-plane resolution = 1.5625 × 1.5625 mm^2^). Anatomical T1-weighted 3D axial spoiled gradient (SPGR) echo sequences (TR/TE = 7.92/2.98 ms; flip angle = 15°; FOV = 256 × 256 mm; slice thickness = 1 mm; in-plane resolution = 1 × 1 mm^2^) were acquired to provide high-resolution structural images.

### fMRI Data Preprocessing

fMRI data preprocessing was performed with SPM5 (Wellcome Department of Imaging Neuroscience, London, UK). It included correction for head movements via realignment of all images to the mean image of the scan using rigid body transformation with six degrees of freedom, normalization of the images to Montreal Neurological Institute (MNI) space by co-registration to the EPI MNI template via affine transformation (re-sliced voxel size was 3 × 3 × 3 mm^3^), and spatial smoothing of the data with a 6 mm FWHM. Participants’ head motion displayed a framewise displacement (FD; Power et al., 2012) averaging at FD = 0.085 ± 0.047, with three participants having 0.20 < FD < 0.26. There was no difference in FD between the two rs-sessions (FD_rest1_ = 0.085 ± 0.057, FD_rest2_ = 0.085 ± 0.044, *p* = 0.91). The first six images of each functional resting scan were excluded to allow for T2*equilibration effects. Before further analysis, blood oxygenation level-dependent (BOLD) signals were filtered to low frequency fluctuations (0.01–0.08 Hz) using DPARSF toolbox (Chao-Gan and Yu-Feng, 2010).

### fMRI Data Parcellation

We used a previously reported whole brain functional parcellation which was based on the application of correlation-based clustering procedure on rs-fMRI data of healthy subjects that partitions the brain volume into 517 regions or parcels (Craddock et al., 2012). To note, using parcels diminishes the spatial resolution compared to using voxels, yet using information at the voxel level entails a large redundancy in information, while information at the level of parcel has been demonstrated as reliable. Moreover, this procedure enabled us to focus subsequent analyses on functionally defined distinct brain regions which are less sensitive to noise and are interpreted more easily. Parcels were masked to include gray matter voxels only using the WFU Pick Atlas Tool (Maldjian et al., 2003; Stamatakis et al., 2010) and 54 parcels that had less than five voxels in common with the gray matter mask were excluded, leaving 463 parcels. This ensured that only gray matter voxels were used in the analysis. For each subject, average BOLD value across all gray matter voxels was calculated within each parcel at each time point of the two rest periods. These time series were used as the parcel’s signal. To reduce the effect of physiological artifacts and nuisance variables, six motion parameters, cerebrospinal fluid, and white matter signals were regressed out of these parcel signals.

### fMRI Data Analysis

**Figure 1:**
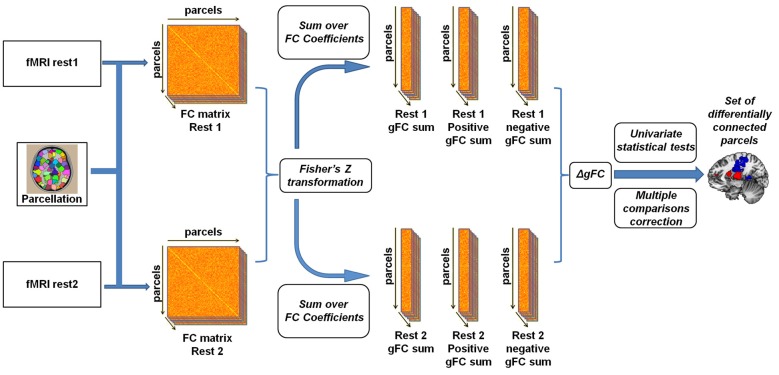
Illustration of the global resting-state functional connectivity (rs-FC) analysis pipeline. Inspired by Cole et al. (2010) and Maron-Katz et al. (2016), following parcellation, cross-correlation matrices were calculated for each subject and each resting-state session resulting in an rs-FC matrix. We subsequently Fisher Z transformed the correlation coefficients and computed the global rs-FC (gFC) of the sum of each node (the sum of correlation of node signals with those of all other nodes). We also separated gFC values into positive-only and negative-only values, resulting in three gFC values per subject per session. Finally, a univariate one-sample *t*-test was conducted on the difference (∆gFC = rest2 − rest1) between each of the three values between rs-sessions applying a multiple comparisons correction.

To examine experimental effects of anger on rs-fMRI, we conducted a parcel-based analysis (Figure 1) inspired by previous such efforts (Cole et al., 2010; Maron-Katz et al., 2016), in which the relationship between each two brain regions was estimated by calculating the Pearson correlation coefficient between their corresponding signals. This was done for each subject and each rs-session separately. Coefficient values were next Fisher Z transformed to fit a normal distribution. We initially tested these coefficients for changes in rs-FC between rest1 (before) and rest2 (after) but no changes in these pairwise rs-FC maps were significant following correction for multiple comparisons using the false discovery rate (FDR) procedure (Benjamini and Yekutieli, 2001). To note, while FDR is a more lenient procedure compared to Bonferroni, it has considerable power advantages especially compared to conservative procedures such as Bonferroni which assume complete independency between tests (an assumption which is clearly violated in the case of fMRI data). Therefore we computed for each parcel and rest period the sum of all the correlation coefficient values with all other parcels. This measure of gFC captures the level of FC a certain brain region has with all other brain regions in the entire brain. Since the sum over all coefficient values holds the risk of positive and negative values cancelling each other out, we also computed the sum of only positive and only negative values separately. Finally, we calculated the change in rs-FC by subtracting gFC level estimates of rest1 from the corresponding estimates in rest2, resulting in three gFC change values (denoted ∆gFC, ∆gFC^+^ and ∆gFC^−^) for each brain region and for each subject. To identify brain regions that demonstrated significant change following the anger induction, we applied a one-sample *t*-test on the three change values of each parcel across all subjects and applied the FDR procedure to account for multiple comparisons. We next performed a secondary analysis in which we examined the changes in all functional connections of the brain regions identified in the previous step with all other brain regions. This step aimed to reveal which specific and more localized connections underlie the differences found in the gFC analysis. FDR procedure was again applied to account for multiple comparisons.

### Volumetric Data Preprocessing and Analysis

The volumetric analysis was performed using the FreeSurfer V5.3 image analysis suite^1^ which is an automated software for brain segmentation based on probabilistic atlas and intensity values. Briefly, the automated procedure includes skull-stripping, intensity normalization, Talairach transformation (the MNI305 template is also used in this processing step; Collins et al., 1994), tissue segmentation, and surface tessellation (Dale et al., 1999; Fischl et al., 1999a,b). The complete FreeSurfer analysis pipeline was performed with manual intervention and quality assurance of the data. Based on the automated segmentation and the fMRI data-driven results we extracted for each subject the right amygdala, right IFG (pars orbitalis) and intra-cranial volumes (mm^3^). We subsequently calculated the adjusted volume of amygdala and IFG by dividing each subjects’ volume by his intra-cranial volume.

## Results

### Data-driven Analysis

**Figure 2:**
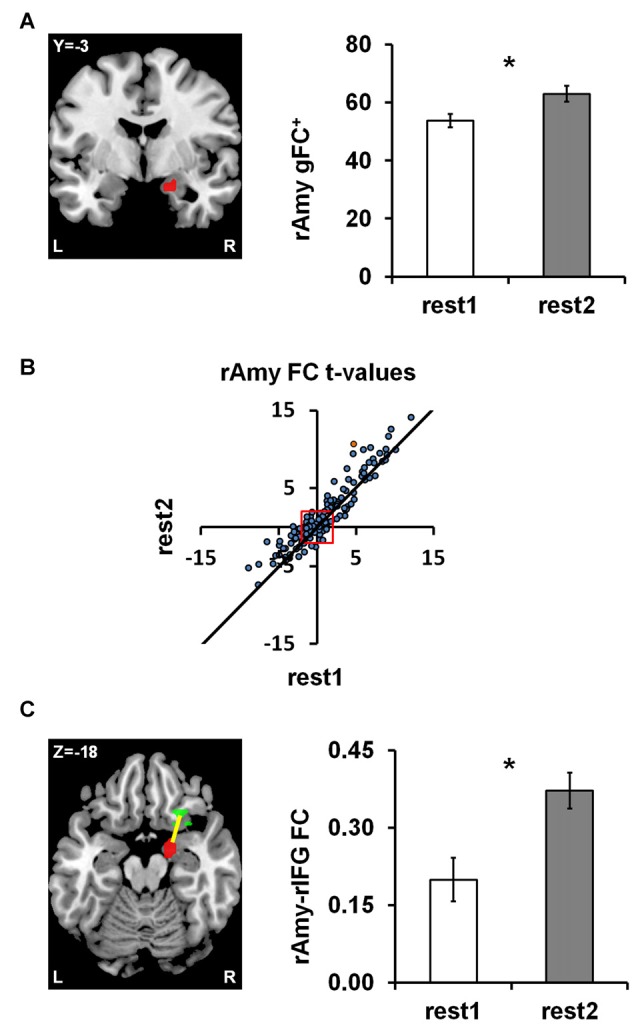
Anger-induced FC modulations. **(A)** A single parcel located in the right medial Amygdala (rAmy; to the left; MNI coordinates: *x* = 18, *y* = −3, *z* = −18) for which global positive FC (gFC^+^) significantly increased between rest1 and rest2 (the extent of change is shown on the right). **(B)** The scatter plot illustrates all 462 amygdala connections per rest1 (x-axis) and rest2 (y-axis) as *t*-values of the across participants FC calculated in comparison to zero. All dots above the diagonal (311 in number) reflect connections that increased between rs-sessions. All dots beyond the red square have significant t-values (*t*_(43)_ = ±2.017, *p* < 0.05), 145 of which had positive FC in both rs-sessions. **(C)** Examining all pairwise FC changes involving the amygdala parcel revealed a single significant change characterized by an increase in FC with a parcel located in the right Inferior Frontal Gyrus (rIFG; *x* = 26, *y* = 23, *z* = −18). The orange dot in **(B)** represents the rAmy-rIFG connection. Error bars indicate standard error of mean, *false discovery rate (FDR) *q* < 0.05, *n* = 44.

To obtain a data driven account of rs-FC modulations following anger induction, we employed the parcel-based univariate gFC analysis. The results revealed a single significant brain region located in a medial region of the right amygdala (31 voxels centered at MNI coordinate: *x* = 18, *y* = −3, *z* = −18) for which positive gFC increased between rest1 (53.70 ± 15.86) and rest2 (62.91 ± 18.32; ∆gFC^+^=9.21 ± 14.51; *t*_(df = 43)_ = 4.21, *p* = 0.0001; FDR *q* < 0.05; Figure 2A). In order to account for the possibility that the sum of all positive FCs may have involved different parcel pairs for each subject, we validated the finding using, for each session, a fixed set of 145 parcels that demonstrated a significant positive FC with the right amygdala in both sessions (Figure 2B). The amygdala ∆gFC^+^ based only on these 145 connections per subject per rs-session similarly showed a significant increase between rest1 (27.37 ± 12.29) and rest2 (33.11 ± 10.94; ∆gFC^+^_145_ = 5.73 ± 9.79; *t*_(43)_ = 3.88, *p* = 0.0003). To note, there was no group differences between civilians and soldiers in the ∆gFC^+^ of the amygdala between before and after anger (*t* = 1.51, *p* = 0.14).

### Amygdala Parcel Analysis

We subsequently examined all 462 functional connections of the right amygdala parcel using a similar univariate analysis as implemented in the initial pairwise FC analysis (see “fMRI Data Analysis” section in “Materials and Methods”), in order to individuate the connections that significantly contributed to the increased ∆gFC^+^ of the amygdala between rs-sessions. We found a single connection with a parcel located in the right IFG pars orbitalis (24 voxels centered at *x* = 26, *y* = 23, *z* = −18) that showed a significant increase between rest1 (0.20 ± 0.28) and rest2 (0.37 ± 0.23; ∆FC = 0.17; *t*_(43)_ = 4.29, *p* = 0.0001; FDR *q* < 0.05; Figure 2C). This increase in connectivity was found also when applying global signal removal by extracting the mean time course over all white matter and CSF voxels and adding this mean to the set of confound variables regressed out of the time course of each parcel (*p* = 0.0135). To note, there was no group differences between civilians and soldiers in the ∆FC of the amygdala-IFG connectivity between before and after anger (*t* = 0.27, *p* = 0.79).

### Correlation Analyses with State Anger Measures

We next tested the relation between the rs-FC changes, namely right amygdala ∆gFC^+^ and right amygdala-right IFG ∆FC, and individual differences in state measures associated with anger induction, namely total-gain and angry feelings during the game, using Spearman’s correlation coefficient with a two-tailed significance test. No significant relationships were found (*p*-values > 0.319).

### Correlation Analyses with Trait Anger Measures

**Figure 3:**
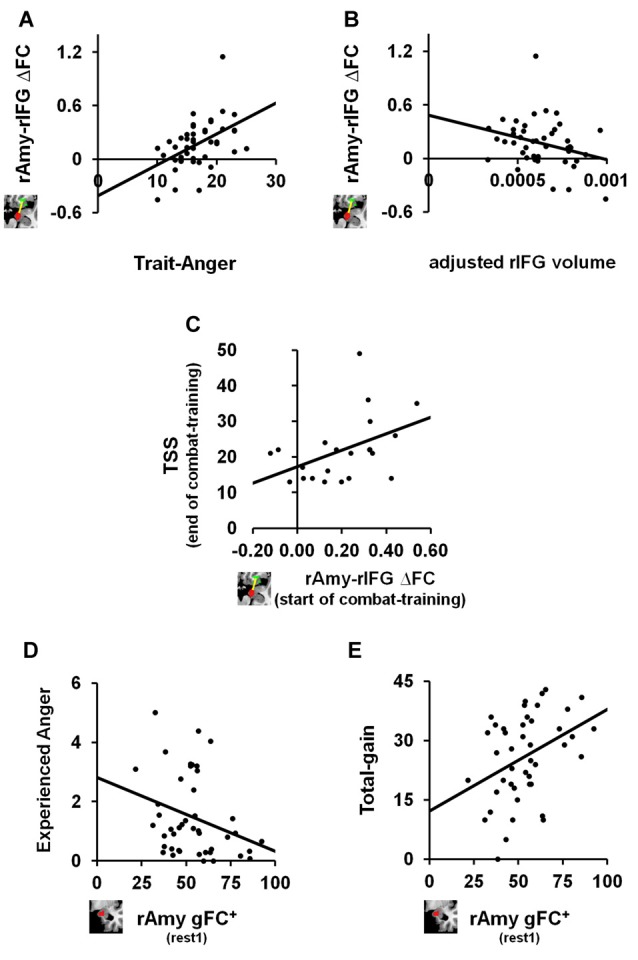
The relation between anger-induced FC modulations, state and trait measures of anger and traumatic stress symptoms (TSS). For trait-like measures, a greater increase in FC between right amygdala and right IFG between before and after the anger-induction (rest2-rest1) correlated **(A)** positively with trait-anger (*ρ* = 0.469, *p* = 0.001, FDR *q* < 0.05; *n* = 44) and **(B)** negatively with the adjusted volume size of the same right IFG (*ρ* = −0.304, *p* = 0.045, uncorrected; *n* = 44). To note, these two correlations remain significant even when removing the outlier: *ρ* =0.443, *p* = 0.003, FDR *q* < 0.05 and *ρ* = −0.313, *p* = 0.041, uncorrected, respectively. For TSS as measured by the PCL-M questionnaire, **(C)** greater increase in FC between right amygdala and right IFG between before and after the anger-induction as measured in soldiers at the beginning of combat-training, predicted more TSS 1 year later at the end of combat-training (*ρ* = 0.459, *p* = 0.036, uncorrected; *n* = 21). Finally, for state measures of anger we found that higher gFC^+^ of the right Amygdala before (rest1) playing an anger-inducing Ultimatum Game predicted **(D)** lower reported feelings of anger experienced during the game (*ρ* = −0.332, *p* = 0.027, uncorrected; *n* = 44) and **(E)** higher total-gain accumulated throughout the game (*ρ* = 0.353, *p* = 0.019, uncorrected; *n* = 44).

We next tested the relation between the identified anger-induced rs-FC modulations and trait-like measures, namely trait-anger, adjusted right amygdala volume and adjusted right IFG volume. We found significant relationships such that higher trait-anger levels and smaller adjusted right IFG volume were associated with a greater increase in right amygdala-right IFG FC between rs-sessions (*ρ* = 0.469, *p* = 0.001, FDR *q* < 0.05; Figure 3A and *ρ* = −0.304, *p* = 0.045, uncorrected; Figure 3B, respectively).

### Correlation Analyses with TSS

Finally, and in view of results thus far, we examined whether right amygdala gFC+ at baseline or right amygdala-right IFG ∆FC between rs-sessions would predict TSS levels among soldiers at the end of a 1 year period of intense combat-training. We found a positive relationship such that a greater increase in right amygdala-right IFG FC following anger induction was associated with higher TSS levels as measured by the PCL-M (*ρ* = 0.459, *p* = 0.036, uncorrected; *n* = 21; Figure 3C).

### *Post hoc* Correlation Analysis

We further explored whether baseline (i.e., rest1) levels of right amygdala gFC^+^ or right amygdala-right IFG FC were associated with state or trait anger measures. We found significant relationships such that more right amygdala gFC^+^ at baseline was associated with less angry feelings (*ρ* = −0.332, *p* = 0.027, uncorrected; Figure 3D) and with more total-gain (*ρ* = 0.353, *p* = 0.019, uncorrected; Figure 3E). To note, we also found a significant relationship such that smaller adjusted right IFG volume was associated with more reported angry feelings during the game (*ρ* = −0.278, *p* = 0.032, uncorrected; *n* = 60).

## Discussion

The current study implemented a data-driven whole-brain analysis to investigate the change in endogenous neural dynamics in the aftermath of an interpersonal anger experience by examining modulations in FC of rs-fMRI. We revealed an increase in positive gFC of the right amygdala, and specifically an increase in rs-FC between the right amygdala and right IFG, following an anger-infused Ultimatum Game. Higher levels of right amygdala positive gFC before the angering game predicted less reported anger and more monetary gain during anger provocation. Moreover, a greater increase in the amygdala-IFG connection in the aftermath of anger was associated with smaller volumes of the right IFG, higher trait-anger levels and with higher TSS among soldiers, measured 1 year later at the end of combat-training. Together, though some correlations did not survive correction for multiple comparisons, these findings potentially link neural dynamics related to the traces of anger with state and trait like characteristics of anger, and with later development of pathological symptomatology.

The amygdala and IFG were recently associated with a neural model of anger (Gilam and Hendler, 2015), whereby the amygdala was suggested to be involved in threat detection and in mediating negative affect and thus subsequently contributing to reactive aggression, while the IFG was attributed a role in regulating anger experience and inhibiting aggressive impulses. We previously demonstrated that adaptive coping with anger during provocation was associated with increased vmPFC activation and increased posterior Insula-medial Thalamus connectivity (Gilam et al., 2015). The increased amygdala-IFG connectivity following anger demonstrated here suggests that different neural processes are engaged when coping with anger in its aftermath compared to during provocation. A recent model postulated a reverse relationship between a brain network involved in salience processing, which includes the amygdala and insula, and a brain network involved in executive control, which includes regions of the PFC, in the dynamics of exposure to and recovery from acute stress (Hermans et al., 2014). Congruent with current findings, an increase in resource allocation to the salience network and the reverse for the executive control was suggested to mediate maladaptive recovery following stress. However, while anger involves stress-related neuro-physiological systems such as the Locus Coeruleus-Noradrenergic system (Gilam et al., 2015), it is not strictly a stress response. The present findings may thus be informative for a broader account on the interaction between emotion generation and regulation processes and their unfolding beyond the immediate emotional experience.

In agreement with a previous study that explicitly instructed participants to ruminate about anger (Fabiansson et al., 2012), we revealed an increase in amygdala-IFG neural coupling in the aftermath of anger. We extend these results by detecting this increase during non-instructed rs-fMRI using a whole-brain data-driven analysis. In fact, our results are concordant with a broader involvement of amygdala and IFG activity and connectivity in rumination, not necessarily specific to anger (Ray et al., 2005; Kross et al., 2009; Hooker et al., 2010; Kühn et al., 2012; Milazzo et al., 2016). Of particular interest, it was demonstrated that high trait-rumination was associated with smaller gray matter volume and overlapping lower neural activations in the right IFG (Kühn et al., 2012). Congruently, we showed that a larger increase in amygdala-IFG rs-FC in the aftermath of an angering experience was associated with lower gray matter volume of the IFG. The increase in amygdala-IFG connectivity also positively correlated with trait-anger which was shown to have a positive association with rumination (Sukhodolsky et al., 2001; Wilkowski and Robinson, 2010). In contrast to this result, it was elsewhere shown that higher trait-anger was associated with lower rs-FC between amygdala and a region in the orbito-frontal cortex, just anterior to the IFG (Fulwiler et al., 2012). However, in that study only one rs-fMRI scan was acquired irrespective of an emotional experience, while here we probed two rs-fMRI scans immediately before and after an interpersonal experience of anger, which might explain this discrepancy. In this respect, we also found that smaller gray matter volume in the IFG, which was previously associated with more trait-rumination (Kühn et al., 2012), was associated with higher anger reported to have been experienced during the anger-infused game, alluding to the general positive association between anger and rumination (Bushman et al., 2005; Pedersen et al., 2011).

Nevertheless, it should be noted as a limitation that while we examined the volume of specific brain regions in anatomical overlap with those regions found in the rs-FC analysis, namely right amygdala and right IFG pars orbitalis, the anatomical labeling of brain regions in the volumetric analysis is approximated to Talairach space (Collins et al., 1994; Dale et al., 1999). This was not used in the rs-FC analysis. Thus said, it was previously demonstrated that the volume calculated by FreeSurfer is comparable to that of other software which calculate brain volume and do not use such an approximation procedure (Tae et al., 2008; Klauschen et al., 2009; Grimm et al., 2015). An additional limitation should be noted regarding the gFC approach that was used. We choose this approach due to the low statistical power of the rs-FC pairwise approach, which did not yield any significant results. Unlike the pairwise approach, gFC is a summary metric which does not capture the whole brain FC at the level of each functional connection. Thus said, to minimize information loss, we calculated positive and negative gFC separately. Future studies able to reveal large-scale network changes following anger could potentially implement graph-theoretical measures to further characterize the underlying properties of these network changes.

While findings of increased amygdala connectivity and specifically with the IFG seem to indicate maladaptive recovery from anger, possibly related to ruminative processing, three main limitations should be cautioned. First, we did not have a direct measure of rumination. Second, we were only able to show an indirect relation between state measures of anger and the identified rs-FC modulations. Namely, while only the amygdala connectivity patterns changed between before and after anger, the relation to angry feelings and monetary gain accumulated in the anger-infused UG was found to amygdala connectivity at baseline, before anger. Therefore, and third, it is possible that a latent variable such as habituation or fatigue contributed to the increase in amygdala-IFG connectivity in the aftermath of anger. Nevertheless, it should also be noted that studies investigating neural dynamics during rs-fMRI following acute stress have similarly shown increased amygdala connectivity (van Marle et al., 2010; Veer et al., 2011), which was also associated with a sustained negative experience and thus with maladaptive coping (Vaisvaser et al., 2013). Elsewhere, positive amygdala-IFG neural coupling was demonstrated to associate with unsuccessful or enhanced efforts to exert cognitive control via reappraisal over an evoked negative emotion (Wager et al., 2008). In contrast, a different study reported that greater amygdala-IFG neural coupling during rs-fMRI was associated with subsequent success in using reappraisal to down regulate negative affect associated with angry faces (Morawetz et al., 2016). We therefore do not know what the amygdala-IFG connectivity reflects *per se*. Ultimately, future studies will hopefully disentangle between the different regulation strategies (implicit/explicit), conditions (rest/task) and timings (before/after emotional experiences) in order to better understand and interpret these findings.

However, we further support that the increase in amygdala-IFG neural coupling following anger associates with maladaptive or dysregulated emotional processing by demonstrating that among soldiers undergoing combat-training, greater increase in amygdala-IFG connectivity at the beginning of combat-training predicted higher levels of TSS at the end of training. Though hyper-reactive amygdala and hypo-reactive IFG are of the most robust and consistent findings in studies comparing PTSD patients with healthy and trauma exposed controls (Hayes et al., 2012; Patel et al., 2012), amygdala-IFG connectivity has yet been demonstrated as a potential predisposing risk-factor on TSS development (Admon et al., 2013). There is a growing number of studies demonstrating altered rs-FC in PTSD patients (Peterson et al., 2014), several of which have shown weaker positive amygdala-IFG connectivity compared to control groups (Sripada et al., 2012; Brown et al., 2014; Zhang et al., 2016). Though seemingly incongruent with current results, unlike those previous studies, we examined rs-FC changes in the aftermath of anger, emphasizing neural processing associated with emotional recovery and possibly related to rumination. Indeed, rumination was found as a powerful predictor of persistent stress symptomatology (Michael et al., 2007) and in fact also as a mediator between TSS and anger (Orth et al., 2008). Though previous findings provided a neural link between anger and TSS (Gilam et al., 2017; Lin et al., 2017), the results found here provide a first such link between emotional coping in the aftermath of anger and stress related symptomatology. Nevertheless, our findings necessitate additional inquiry since they were limited by the relatively small sample size which consisted of males only, and that soldiers did not experience actual traumatic events and evidenced moderate levels of TSS. In this regard it should be noted as a limitation that this result as well as several other significant correlations did not survive for correction of multiple comparisons.

In conclusion, the increase of amygdala-IFG FC found here seems to be associated with maladaptive processing in the aftermath of anger and thus may serve as a potential target for understanding and treating psycho-pathological conditions characterized by excessive anger and/or emotion dysregulation such as anxiety, depression, and personality disorders.

## Author Contributions

GG designed the experiment, collected the data, performed behavioral and integration analyses, wrote the original draft and reviewed and edited the manuscript. AM-K preprocessed and analyzed resting-state fMRI data and reviewed and edited the manuscript. EK preprocessed and analyzed anatomical data. TL designed the experiment and collected the data. EF provided resources and collected the data. RS supervised resting-state analysis and reviewed and edited the manuscript. TH designed the experiment, funded and supervised the project and reviewed and edited the manuscript.

## Conflict of Interest Statement

The authors declare that the research was conducted in the absence of any commercial or financial relationships that could be construed as a potential conflict of interest.

## Footnotes

^1^http://surfer.nmr.mgh.harvard.edu/
